# New Trends in Treating Cushing’s Disease

**DOI:** 10.17925/EE.2024.20.2.3

**Published:** 2024-04-08

**Authors:** Odysseas Violetis, Krystallenia I Alexandraki

**Affiliations:** 2nd Department of Surgery, Aretaieio Hospital, National and Kapodistrian University of Athens, Athens, Greece

**Keywords:** Cushing’s disease, immunotherapy, levoketoconazole, novel drugs, osilodrostat, pasireotide, relacorilant

## Abstract

Rates of recurrence and/or persistence of Cushing’s disease after surgical treatment are high. Recently, advances in molecular insights and a better understanding of pathophysiology have enabled the development of potential therapeutic targets that could control adrenocorticotropic hormone (ACTH) and cortisol secretion or even reduce tumour cell proliferation. At the pituitary level, pasireotide is an approved somatostatin receptor ligand, and compounds targeting cell cycle regulation, cell signalling and epigenetics are now under investigation. Levoketoconazole and osilodrostat are novel steroid inhibitors, and relacorilant overcomes the adverse effects of mifepristone. Adrenal ACTH receptor blockage and immunotherapy could also play a role.

Cushing’s disease (CD) is caused by an adrenocorticotropic hormone (ACTH)-secreting pituitary adenoma, or rarely carcinoma, and is considered a highly morbid endocrine disorder with few medical options.^1,2^ Although transsphenoidal pituitary surgery (TSS) is the mainstay of treatment for this disease, up to one-third of the patients eventually relapse and are required to be treated with a variety of drugs, as none can achieve optimal disease control.^3,4^ Drugs may aim directly at the corticotroph adenoma (pasireotide and cabergoline), act on the adrenal steroidogenesis pathway mitigating the cortisol synthesis (such as ketoconazole, etomidate, metyrapone and mitotane) or block the glucocorticoid receptor (mifepristone). Even though the first category of drugs is deemed more rational as they act directly on the pituitary tumour, targeting the source of ACTH secretion aiming to control or shrink the tumour, 60–75% of patients are insensitive to current pituitary-targeted agents.^5^ Furthermore, use of classic adrenal-directed drugs can achieve control of hypercortisolism in more than half of patients, but may induce severe adverse effects.^6,7^ Specifically, hepatotoxicity is a major issue in treatment with ketoconazole, while hirsutism and hypertension are associated with metyrapone. Therefore, developing new drugs that are well tolerated and effective in stabilizing the disease is considered pivotal.^8^

Although ACTH-secreting tumours express somatostatin receptor 2 (SSTR2) and SSTR5 messenger RNAs (mRNAs) (>85%) and, to a lesser degree, SSTR1 mRNA (63%), in the membrane, SSTR5 is predominant.^9^ Although the precise mechanism remains unclear, it seems glucocorticoids downregulate SSTR2, leaving SSTR5 intact, explaining the lack of efficacy of traditional SSTR2-targeting analogues in patients with CD.^10–12^ Pasireotide is a novel somatostatin analogue targeting four out of five SSTR subtypes, with the highest affinity for SSTR5.^3–5,10^ The first randomized phase III clinical trial (Safety and Efficacy of Different Dose Levels of Pasireotide in Patients With de Novo, Persistent or Recurrent Cushing's Disease; ClinicalTrials.gov identifier: NCT00434148) involving 162 patients with CD treated with subcutaneous pasireotide showed urine-free cortisol (UFC) normalization at month 6 in 15–26% of the patients without uptitration, and, simultaneously, a significant clinical improvement was noted in most patients.^13,14^ However, it seems that pasireotide was more efficacious in cases with mild hypercortisolism. The response was rather rapid and sustained over a longer period, allowing the possibility of predicting how patients will respond to treatment early after the treatment initiation. Subsequent sustained improvement in blood pressure and lipid profile, as well as in quality of life and visceral adiposity, was noted during the long-term treatment with pasireotide.^15^ Notably, adiposity and blood pressure were ameliorated, even if complete biochemical control was not achieved.^16^ Similarly, tumour shrinkage was noticed irrespective of UFC reduction, distinguishing the action of pasireotide on tumour size and the inhibition of secretion.^17^ To avoid subcutaneous pasireotide administration twice daily, a long-acting release formulation was developed and confirmed to have similar efficacy and safety to the short-acting form.^18^ Despite the benefits of pasireotide, about two-thirds of the patients developed hyperglycaemia-related adverse events, requiring additional antidiabetic treatment.^14,17,18^

Several centrally acting agents are in development (Figure 1, Table 1).^13,19–28^ First, by binding to the retinoic acid receptor and retinoid X receptor, retinoid acid modulates proopiomelanocortin (POMC) expression, and studies on AtT20 mice pituitary ACTH-secreting tumour cells and animal models have shown that it can effectively suppress ACTH secretion and adenoma growth.^29^ In two prospective studies on patients with CD, retinoic acid achieved UFC normalization and clinical improvement in a significant proportion of patients with mild hypercortisolism. Regarding its safety, the most common adverse effects were transient conjunctival irritation, cheilitis, mucositis, nausea, headache and arthralgias.^27,28^

**Table 1:** Drugs for the management of Cushing's disease tested in clinical studies^13,19–28^

Drug name	N	Duration	Effectiveness	Side effects	Clinical trial (phase, ClinicalTrials.gov identifier)
Levoketoconazole	94 (80 CD)	Dose-titration period (2–21 weeks) and a 6 month maintenance period	29/94 (31%) UFC normalization without dose increase.	Gastrointestinal disturbances, headache, edema, liver enzyme increase , adrenal insufficiency	SONICS (phase III; NCT01838551)^19^
			34/55 (62%) and 9/55 (16%) had a complete and a partial response irrespective of dose increase (at the end of the core study), respectively		
	84 (70 CD)		Open-l abel titration-maintenance (14–19 weeks) followed by double-blind, randomized-withdrawal (~8 weeks) and restoration (~8 weeks) phases		72.2 % (57/79) achieved a complete or a partial response (at the end of titration-maintenance period).	LOGICS (phase III; NCT03277690)^20^
					Normalization of mUFC in 50.0% patients in the levoketoconazole group versus 4.5 % in the placebo group (at the end of the randomization phase).	
					Loss of mUFC response was significantly higher in the placebo group compared to levoketoconazole group (40.9% versus 95.5 %)	
Osilodrostat	137 CD	48 weeks (four phases)	66 % had a complete response and 9 % had a partial response at the end of the core study (week 48)	Fatigue, nausea, headache, diarrhea, adrenal insufficiency hypertension, hypokalaemia, QT-interval and in females hirsutism and acne	LINC3 (phase III; NCT02180217)^21^
	73 CD	48 weeks (12 week, randomized, double-blind, placebo-controlled period and a 36 week, open-label period)	68.5 % had a complete response and 11.0 % had a partial response at the end of the core study (week 48)		LINC4 (phase III; NCT02697734)^22^
Relacorilant	35 (23 CD)	20 weeks (up-titration period of 12–16 weeks and a 4 week, open-l abel, stable-dose period)	In the low dose group, 42% and 15% demonstrated a BP reduction of ≥5 mmHg and a glycaemic reduction respectively.	Back pain, headache, oedema, nausea, pain at extremities, diarrhea and dizziness	Phase II study (NCT02804750)^23^
			In the high dose group, 64% and 50% demonstrated a BP reduction of ≥5 mmHg and a glycaemic reduction respectively		
Pasireotide	162 CD	12 months	Complete response in 15% and 26% and partial response in 18% and 13% in low and high dose group respectively (month 6)	Diarrhea, cholelithiasis, hyperglycaemia, fatigue, abdominal pain, nausea	Phase III study (NCT00434148)^13^
Pasireotide LAR	150 CD	12 months	Complete response in 41.9% and 40.8% and partial response in 5% and 13% in low and high group respectively (month 7)		Phase III trial (NCT01374906)^24^
Roscovitine(seliciclib)	9 CD	4 weeks	5 patients had near or >50% UFC reduction. None achieved normalization	Transient elevated liver enzymes, anemia and elevated creatinine levels	Phase II (NCT02160730, NCT03774446)^25,26^

Tretinoin	7 CD	12 months	3 patients had normalization or >50% UFC reduction (month 12)	Conjunctival irritation, nausea, headache and arthralgia	Prospective, multicenter study^27^
Isotretinoin	16 CD	12 months	4 patients had UFC normalization (month 12)	Conjunctival irritation, cheilitis, mucositis, nausea, headache, and arthralgias	Prospective open trial^28^

*Complete response: UFC normalization; partial response: ≥50% UFC reduction.*

*BP = blood pressure; CD = Cushing's disease; LAR = long-acting release; mUFC = mean urinary free cortisol; UFC = urinary free cortisol.*

**Figure 1:**
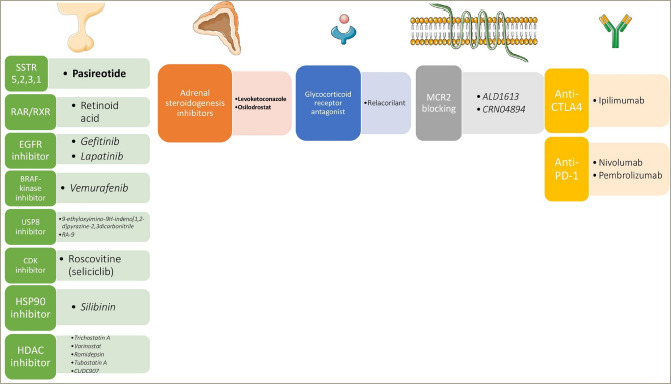
The main mechanism of action of the novel drugs for Cushing’s disease

*Approved drugs are given in bold, drugs tested in humans but not approved are given in non-bold and drugs tested only* in vitro *or* *in vivo in animals are given in italics. Graphical images of human body parts and molecules were sourced at https://smart.servier.com, which are available under a CC BY 4.0 International license (https://creativecommons.org/licenses/by/4.0/).*

*BRAF = B-raf proto-oncogene, serine/threonine kinase; CDK = cyclin-dependent kinase; CTLA4 = cytotoxic T-lymphocyte associated protein 4; EGFR = epidermal growth factor receptor; HDAC = histone deacetylase; HSP90 = heat shock protein 90; MCR2 = melanocortin 2 receptor; PD-1 = programmed death-1; RAR = retinoic acid receptor; RXR = retinoid X receptor; SSTR = somatostatin receptor; USP8 = ubiquitin specific peptidase 8, RA-9: (3E,5E)-3,5-Bis[(4-Nitrophenyl)methylene]-4-piperidinone.*

More than half of pituitary tumours, including corticotroph adenomas, express epidermal growth factor receptor (EGFR), which plays a key role in POMC expression. Interestingly, 36–62% of corticotroph adenomas show gain-of-function mutations in the gene encoding for *USP8*.^30,31^ This gene codes for a protein with deubiquitinase activity, which rescues proteins such as EGFR from lysosomal degradation. Mutated *USP8* exerts higher deubiquitinase activity, probably explaining the aberrant EGFR signalling in corticotrophs.^31^ To date, two EGFR tyrosine kinase inhibitors, gefitinib and lapatinib, have been evaluated for the treatment of pituitary tumours.^32^ *In vitro* and *in vivo* studies have shown that both could significantly decrease POMC and ACTH productionm as well as reduce proliferation activity and tumour size.^33,34^ An ongoing clinical trial is evaluating gefitinib in patients with *USP8*-mutated CD, but no results released till now (Targeted therapy with gefitinib in patients with USP8-mutated Cushing's disease; ClinicalTrials.gov identifier: NCT02484755).^35^ It is estimated that 16.5% of *USP8* wild-type corticotroph adenomas carry *BRAF* (B-raf proto-oncogene, serine/threonine kinase) mutations encoding *p.V600E*, resulting in increased MAPK (mitogen-activated protein kinase) activity and subsequent POMC expression. A BRAF-kinase inhibitor, vemurafenib, was reasonably evaluated, and it was discovered to suppress ACTH secretion from primary cultures of human corticotroph adenomas harbouring *BRAF V600E*.^36^ Apparently, except for tyrosine kinase inhibitors, specific *USP8* inhibitors are being studied. Of note, POMC may be subject to ubiquitination, independent of *USP8* sequence status, thereby indicating the use of *USP8* inhibitors regardless of *USP8* mutation.^37^ Reduced ACTH secretion and cell proliferation and increased apoptosis were noted with 9-ehtyloxyimino-9H-i ndeno[1,2-b] pyrazine-2,3dicarbonitrile and RA-9 9 ([3E,5E]-3,5-Bis[(4-Nitrophenyl) methylene]-4-piperidinone) *in vitro*.^38,39^

Furthermore, cyclin E is upregulated in corticotroph tumours, and the activation of the cyclin E-cyclin-dependent kinase 2 complex promotes G1-to-S cycle-phase advance and cell division in corticotroph pituitary cells, causing autonomous ACTH overproduction.^40^ Recently, it has been suggested that roscovitine, an inhibitor of cyclin-dependent kinase 2 and cyclin E, may be an effective target medication in corticotroph tumours. In a phase II study on patients with CD (Treatment of Cushing's Disease With R-roscovitine, ClinicalTrials.gov identifier: NCT02160730; and Multicenter Study of Seliciclib (R-roscovitine) for Cushing Disease, ClinicalTrials.gov identifier: NCT03774446), 400 mg twice daily of roscovitine was administered for 4 consecutive days each week for 4 weeks.^25,26^ About 50% of the patients achieved a near or ≥50% reduction in UFC and, simultaneously, exhibited a slightly greater drop in circulating ACTH, advocating the pituitary-directed mechanism of action of roscovitine. Transiently elevated liver enzymes, anaemia and elevated creatinine levels were the most common adverse features.^41^

Heat shock protein 90 (HSP90) is a chaperone protein that induces conformational changes in the glucocorticoid receptor, among others, and affects its transcriptional activity.^42^ Corticotroph adenomas overexpress HSP90, resulting in increased binding to the glucocorticoid receptor, thereby inhibiting its translocation and subsequent DNA binding. Therefore, HSP90 plays a significant role in controlling POMC expression. Overexpression of HSP90 contributes to the partial resistance of tumour cells to glucocorticoids. For that reason, an HSP90 inhibitor may dissociate the glucocorticoid receptor from HSP90, increasing the negative feedback of glucocorticoids at the pituitary level and modulating ACTH secretion.^43^ Silibinin is a C-terminal inhibitor of HSP90 that has been studied as a potential treatment for several malignancies, such as prostate cancer, breast cancer, hepatic cell cancer and lymphoblastic leukaemia, and has a sufficient safety profile.^44,45^ It seems that silibinin inhibits POMC expression and ACTH production while enhancing dexamethasone-induced suppression *in vitro* and *in vivo* in xenografted mice.^45^

Epigenetics is an emerging field of cancer research and drug investigation. That being said, histone deacetylase (HDAC) inhibitors are compounds with promising antineoplastic properties.^46^ Generally, in cancer, acetylation of chromatin is disrupted, and HDAC inhibition might lead to increased expression of tumour suppressor genes and reduced expression of oncogenes. HDAC inhibitors have been shown to inhibit ACTH synthesis, block cell proliferation or modulate glucocorticoid signalling. Trichostatin A, vorinostat, romidepsin, tubastatin A and CUDC907 (fimeprinostat), a dual HDAC and phosphoinositide-3-kinase inhibitor, have shown promising results in experiments.^47^

Next, researchers considered blocking the ACTH receptor in the adrenal glands, called melanocortin 2 receptor (MCR2). ALD1613 (Alder BioPharmaceuticals, now Lundbeck Seattle Biopharmaceuticals, Bothell, WA, USA) is a novel long-acting monoclonal antibody that neutralizes MCR2, reducing intracellular cyclic adenosine monophosphate (cAMP) *in vitro* and *in vivo* in rodents and cynomolgus monkeys. Interestingly, plasma cortisol levels remained low for long after the treatment discontinuation.^48^ A clinical trial examining CRN04894, an oral nonpeptide selective antagonist for ACTH acting at the MC2R, is underway (A study to evaluate the safety and PK of CRN04894 for the treatment of Cushing's syndrome; ClinicalTrials.gov identifier: NCT05804669).^49^ Preclinical data both in rats, in which osmotic pumps of continuous ACTH administration were implanted, and in pituitary corticotrope tumour (AtT-20)-bearing mice have demonstrated robust suppression of corticosterone after daily administration of the oral ACTH antagonist, CRN04894.^50^

Evidence for immune checkpoint inhibitor (ICI) efficacy is emerging from studies on other cancers.^51^ The presence of tumour-i nfiltrating lymphocytes and other mononuclear cells, the discovery of increased levels of programmed death ligand-1 (PD-L1) on the surface of functioning adenomas and the secondary hypophysitis caused by immunotherapy suggest the use of ICIs in the treatment of aggressive corticotroph adenomas and carcinomas resistant to conventional chemotherapies.^52^ Although both combined therapy with ipilimumab (anti-CTLA4 [cytotoxic T-l ymphocyte associated protein 4]) and nivolumab (anti-PD-1), and monotherapy with pembrolizumab (anti-PD-1), have been described only in case reports, they appear to have a promising potential for treating refractory cases.^53,54^ Results from the two registered clinical trials (Nivolumab and ipilimumab in treating patients with rare tumors, ClinicalTrials.gov identifier: NCT02834013; and Nivolumab and ipilimumab in people with aggressive pituitary tumors; ClinicalTrials.gov identifier: NCT04042753) are anticipated to elucidate the efficacy of ICIs.^53–56^

Contrary to the centrally active drugs that, in the majority, are in an experimental stage, steroidogenesis inhibitors show more concrete evidence that they can ameliorate hypercortisolism. Two prospective studies (Treatment for endogenous Cushing's syndrome [SONICS], ClinicalTrials.gov identifier: NCT01838551; and A study to assess the safety and efficacy of levoketoconazole in the treatment of endogenous Cushing's syndrome [LOGICS], ClinicalTrials.gov identifier: NCT03277690) examined levoketoconazole, a ketoconazole enantiomer (2S, 4R), as a possible therapy in patients with Cushing’s syndrome (including those with CD).^19,20^ Starting at a dose of 300 mg/day and titrated up to 1,200 mg/day, levoketoconazole normalized mean urinary free cortisol (mUFC) levels in more than 50% of the patients. Of note, in about 90% of patients with CD, tumour size stayed stable, while no new magnetic resonance imaging (MRI) findings were detected. An improvement in symptoms and comorbidities of Cushing’s syndrome after levoketoconazole treatment was seen, namely in body mass index (BMI), glucose metabolism, even in patients with diabetes mellitus, total cholesterol and low-density lipoprotein (LDL), quality of life, depressive status, peripheral oedema and hirsutism and acne in female patients. As anticipated, hypokalaemia and hypertension were frequent, while liver enzyme derangements and adrenal insufficiency were also reported.^57–60^

Osilodrostat is a potent inhibitor of 11β-hydroxylase and aldosterone synthase, the enzymes responsible for the final step of cortisol and aldosterone biosynthesis, that showed promise as a potent steroid inhibitor based on a prospective double-blind phase III study (Safety and efficacy of LCI699 for the treatment of patients with Cushing's disease; ClinicalTrials.gov identifier: NCT02180217 [LINC3]), including 137 patients with persistent or recurrent CD.^21^ At the end of the core study (at week 48), osilodrostat resulted in a complete response in 66% and a partial response (≥50% reduction of UFC) in 9% of the enrolled patients, respectively. Clinical improvement was also noted by week 48 in body weight, blood pressure, fasting plasma glucose and lipid profile, as well as quality of life and depression status. With regard to tumour size, a similar percentage of patients had either a decrease or an increase of 20% or more in the adenoma (30% versus 29% at week 24 and 38% versus 33% at week 48, respectively), suggesting that osilodrostat did not adversely affect the tumour size.^61^ Noteworthy, a long-term study suggested that up to week 72, osilodrostat succeeded in normalizing UFC in 81% of patients with sustained clinical improvement.^62^ In the phase III multicentre trial LINC4 (Efficacy and safety evaluation of osilodrostat in Cushing's disease; ClinicalTrials.gov identifier: NCT02697734), at the end of randomization, osilodrostat achieved 77.1% UFC normalization versus 8% in the control group.^22^ Blood pressure, lipid and glucose metabolism, adiposity, physical appearance and quality of life were also improved by week 12 and continued up to the end of the core study.^63^ Regarding the safety profile, osilodrostat was generally well tolerated, but in females, hirsutism and acne were reported; hence, women should be monitored for symptoms of hyperandrogenism.^64^

The non-selective steroid inhibition by mifepristone and its related adverse features resulted in the development of a highly selective, non-steroidal modulator of the glucocorticoid receptor called relacorilant.^65^ Unlike mifepristone, relacorilant does not bind to the progesterone receptor, rendering patients free of progesterone receptor modulator-associated endometrial changes, namely endometrial thickening and vaginal bleeding.^66^ A phase II study (Study to evaluate CORT125134 in participants with Cushing's syndrome; ClinicalTrials.gov identifier: NCT02804750) over 20 weeks showed that, after dose stabilization, 42% and 15% of the patients coming from the low-dose group (200 mg/day) demonstrated a blood pressure reduction of ≥5 mmHg and a glycaemic reduction, respectively, whereas in the high-dose group (400 mg/day), an improvement in hypertension and glucose metabolism was achieved in 64% and 50%, respectively.^23,67^ Two phase III clinical trials (A study of the efficacy and safety of relacorilant in patients with endogenous Cushing syndrome [GRACE], ClinicalTrials.gov identifier: NCT03697109; and Efficacy and safety of relacorilant in patients with cortisol-secreting adrenal adenomas [GRADIENT] ClinicalTrials.gov identifier: NCT04308590) testing relacorilant are underway.^68–70^ Interestingly, it has been reported to induce tumour shrinkage in some patients with CD with pituitary macroadenomas. A hypothesis for this finding could be the increased sensitivity to endogenous somatostatin due to the upregulation of SSTR2 on the tumour surface, through which hypercortisolaemia downregulates. It remains to be formally elucidated by the ongoing trials.^71,72^

The management of recurrent or persistent corticotroph adenoma requires an individualized approach by an experienced multidisciplinary team.^73^ Repeat TSS has low remission rates, and radiation therapy has a slow onset of action and can cause severe complications, such as hypopituitarism.^74^ Research on the pathogenesis of corticotroph adenomas has opened new avenues for discovering and developing new treatment options. Although *in vitro* and *in vivo* studies in xenograft mice have shown encouraging results, these compounds remain to be tested in clinical studies. Levoketoconazole and osilodrostat have been approved for controlling cortisol excess and comorbidities. The somatostatin analogue pasireotide is the only currently approved tumour-targeted therapy. It has already been proposed that ACTH-producing adenomas were categorized based on *USP8* mutations, emphasizing the importance of genetics in therapy.^75^ Molecular classification could lead to a more personalized treatment in the future. The selection of the agent depends not only on individual patients’ and tumour characteristics, availability and cost, but also on highly specific molecular patterns.
